# Jammed Pickering Emulsion Gels

**DOI:** 10.1002/advs.202409678

**Published:** 2024-11-14

**Authors:** Jia Zhang, Yuan Zheng, Baoling Guo, Dongpeng Sun, Yao Xiao, Ze Yang, Rongrong Liu, Jingyi Chen, Baiheng Wu, Peng Zhao, Jian Ruan, David A Weitz, Dong Chen

**Affiliations:** ^1^ Department of Medical Oncology The First Affiliated Hospital School of Medicine Zhejiang University Hangzhou Zhejiang 310003 P. R. China; ^2^ College of Energy Engineering and State Key Laboratory of Clean Energy Utilization Zhejiang University Hangzhou Zhejiang 310003 P. R. China; ^3^ Department of Oncology Longyan First Affiliated Hospital of Fujian Medical University Longyan Fujian 364000 P. R. China; ^4^ John A. Paulson School of Engineering and Applied Sciences Harvard University Cambridge MA 02138 USA; ^5^ College of Material Chemistry and Chemical Engineering Key Laboratory of Organosilicon Chemistry and Material Technology Ministry of Education Hangzhou Normal University Hangzhou 311121 P. R. China; ^6^ Zhejiang Key Laboratory of Smart Biomaterials College of Chemical and Biological Engineering Zhejiang University Hangzhou Zhejiang 310027 P. R. China

**Keywords:** colloidal surfactant, drug delivery, emulsion gel, interfacial engineering, Pickering emulsion

## Abstract

Emulsion gels with specific rheological properties have widespread applications in foods, cosmetics, and biomedicines. However, the constructions of water‐in‐oil emulsion gels are still challenging, due to the limited interactions available in the continuous oil phase. Here, a versatile strategy is developed to prepare a new type of emulsion gels, called Jammed Pickering emulsion gels (JPEGs). In the JPEG system, SiO_2_ NPs in the oil phase serve as colloidal surfactants to stabilize water‐in‐oil Pickering emulsions, while positively‐charged NH_2_‐PEG‐NH_2_ molecules in the water phase cross‐link negatively‐charged SiO_2_ NPs at the water/oil interface, making NP‐stabilized water droplets hard to deform and thus jamming the emulsion system to form emulsion gels. The strategy to prepare JPEGs is versatile and applicable to diverse oil phases. The designed JPEGs possess many advantages, including good biocompatibility for widespread applications, shear‐thinning rheological properties for easy processing, good stability Over a wide temperature range and Against centrifugation, good adhesion to wet tissues for tissue engineering, and well‐controlled sustained release Under intestinal conditions. The developed JPEGs are demonstrated to be a promising delivery platform and the strategy to achieve JPEGs will trigger more innovations of material design.

## Graphical abstract

A versatile strategy has been developed to prepare water‐in‐oil emulsion gels, called Jammed Pickering emulsion gels (JPEGs). The designed JPEGs possess good biocompatibility, good stability, good adhesion to wet tissues, and well‐controlled sustained release, which can be a promising delivery platform and the strategy to achieve JPEGs will trigger more innovations of material design.

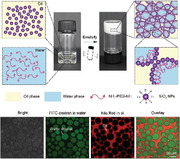

## 1 Introduction

Emulsion gels, which contain both water and oil phases, are soft matters, which combine the benefits of both emulsions and gels.^[^ ^1^ ^]^ In daily life, many systems are made of emulsions and emulsion gels have widespread applications in areas, such as foods,^[^ ^2^ ^]^ cosmetics,^[^ ^3^ ^]^ biomedicines^[^ ^4^ ^]^ and so on.^[^ ^5^ ^]^ Emulsion gels generally have a good storage stability and tunable viscoelastic properties for easy processing, making them superior to emulsions alone.^[^ ^6^ ^]^ For example, emulsion gels could be developed into injectable delivery systems, which could deliver hydrophilic and hydrophobic drugs simultaneously and control their release profiles.^[^ ^7^ ^]^

Traditionally, emulsions are stabilized by molecular surfactants, which could effectively lower the water/oil interfacial tension.^[^ ^8^ ^]^ However, due to the dynamic adsorption and desorption of molecular surfactants at the interface, coalescence and Ostwald ripening will eventually lead to the phase separation of emulsions during their long‐term storage.^[^ ^9^ ^]^ An effective way to solve the stability problem is to replace molecular surfactants with colloidal surfactants,^[^ ^10^ ^]^ which irreversibly adsorb at the water/oil interface, forming Pickering emulsions.^[^ ^11^ ^]^ Silica (SiO_2_) nanoparticles (NPs), whose size and surface properties could be well controlled,^[^ ^12^ ^]^ are the most widely used colloidal surfactants.^[^ ^9^, ^13^ ^]^ So far, the performances of NPs,^[^ ^14^ ^]^ NPs‐surfactants,^[^ ^12^, ^15^ ^]^ NPs‐polymers^[^ ^16^ ^]^ and NPs‐NPs^[^ ^17^ ^]^ in stabilizing Pickering emulsions have been extensively investigated.^[^ ^18^ ^]^

Though NPs could solve the stability problem of emulsions,^[^ ^19^ ^]^ it is still challenging to construct emulsion gels, especially water‐in‐oil emulsion gels. While oil‐in‐water emulsion gels could be achieved by tuning the viscoelastic property of the continuous water phase with the aid of polymers dissolved in water, such as carbomer,^[^ ^20^ ^]^ water‐in‐oil emulsion gels require delicate design, since it is hard to tune the viscoelastic property of the continuous oil phase. Alternatively, interactions between water droplets are introduced into the system to achieve water‐in‐oil emulsion gels.^[^ ^6^, ^21^ ^]^ For example, attractive interactions between NP‐stabilized water droplets are introduced by bridging negatively‐charged water droplets through positively‐charged telechelic polymers, thus forming attractive Pickering emulsion gels (APEGs).^[^ ^6a^ ^]^ In addition to attractive interactions, steric repulsive interactions between NP‐stabilized water droplets could also lead to Pickering emulsion gels. The jamming and thus gelling of NP‐stabilized emulsions typically occurs when the volume ratio of the dispersed phase exceeds 80 vol% as in high internal phase emulsions (HIPEs).^[^ ^22^ ^]^ Despite of the advances, a general strategy to prepare water‐in‐oil Pickering emulsion gels, which are applicable to different scenarios, is still challenging and innovations of novel strategies are highly desired.

In this study, a new strategy is developed to prepare emulsion gels by jamming NP‐stabilized water droplets in the continuous oil phase. In the emulsion gel system, water‐in‐oil emulsions are stabilized by SiO_2_ NPs previously dispersed in the oil phase. Meanwhile, negatively‐charged NPs anchored at the water/oil interface are cross‐linked by positively‐charged NH_2_‐PEG‐NH_2_ molecules previously dissolved in the water phase, making NP‐stabilized water droplets hard to deform and thus jamming the system to form emulsion gels, i.e., JPEGs. JPEGs behave as elastic gels under stationary and could flow under strong shearing, showing typical shear‐thinning behaviors and making them ideal for direct 3D printing in air and water. JPEGs are stable over a wide temperature range and against centrifugation. Because the whole system is biocompatible and the method is versatile, JPEGs could be developed into delivery vehicles with hydrophobic model drugs loaded in the oil phase and hydrophilic model drugs loaded in the water phase. JPEGs also show a good adhesion to wet tissues and encapsulated cargos could be released in a sustained manner in simulated intestinal liquids, providing a promising drug delivery platform for intestinal diseases.

## 2 Results and Discussion

### 2.1 Design and Preparation of Jammed Pickering Emulsion Gels

**Figure 1:**
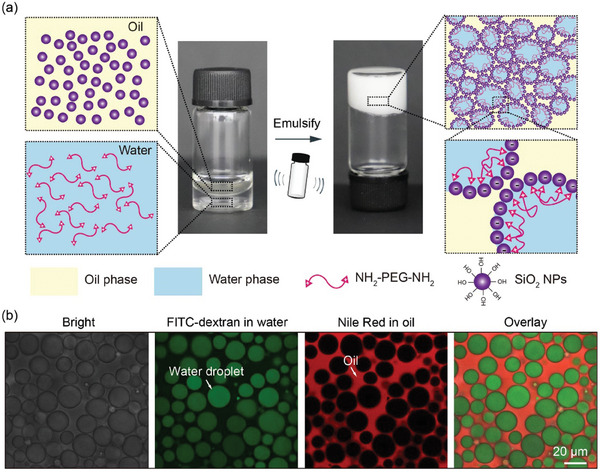
Design of jammed Pickering emulsion gels (JPEGs). (a) During the emulsification process, the water phase is sheared into small droplets, which are stabilized by SiO_2_ NPs, forming Pickering emulsions. Meanwhile, each positively‐charged NH_2_‐PEG‐NH_2_ could bind with two negatively‐charged SiO_2_ NPs at the interface to form a cross‐linked network, making NP‐stabilized water droplets hard to deform and eventually forming JPEGs. (b) Optical and fluorescent confocal microscope images of JPEGs showing that NP‐stabilized water droplets are slightly deformed under steric repulsion. If not specified, the water and oil phases of JPEGs are labeled with FITC‐dextran and Nile Red, respectively, for fluorescent imaging.

To prepare jammed Pickering emulsion gels (JPEGs), hydrophobic SiO_2_ NPs are dispersed in the oil phase, and polyoxyethylene bis(amine) (NH_2_‐PEG‐NH_2_) is dissolved in the water phase. When the oil and water phases are emulsified, the water phase is sheared into small droplets, which are stabilized by SiO_2_ NPs in the oil phase, forming Pickering emulsions. Meanwhile, each positively‐charged NH_2_‐PEG‐NH_2_ could bind with two negatively‐charged SiO_2_ NPs at the interface to form a cross‐linked network, making NP‐stabilized water droplets hard to deform and eventually forming JPEGs, as schematically illustrated in **Figure** **1**a. In the JPEG system, water droplets are closely packed, as shown by the optical and fluorescent images of Figure 1b, and the Pickering emulsions are not flowable, since NP‐stabilized water droplets will impose steric repulsion to neighboring droplets to resist deformation.

### 2.2 Interfacial Property of JPEGs

**Figure 2:**
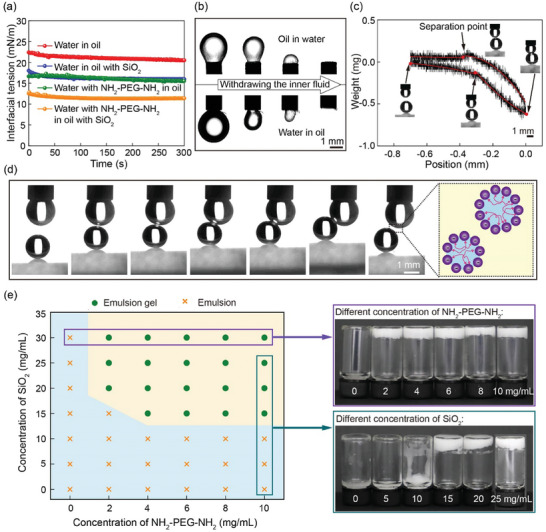
Interfacial properties of JPEGs. (a) Dynamic interfacial tensions of water in oil, water in oil with 20 mg mL^−1^ SiO_2_, water with 10 mg mL^−1^ NH_2_‐PEG‐NH_2_ in oil, water with 10 mg mL^−1^ NH_2_‐PEG‐NH_2_ in oil with 20 mg mL^−1^ SiO_2_. (b) Snapshots showing the buckling of the cross‐linked interface when the inner fluid is withdrawn. Negatively‐charged SiO_2_ NPs at the water/oil interface are cross‐linked by positively‐charged NH_2_‐PEG‐NH_2_ via electrostatic interaction. (c) No attractive force is observed when two NP‐stabilized water droplets separate from each other. Insets are the experimental snapshots. (d) NP‐stabilized water droplets are stable against coalescence, compression, and deformation. (e) Phase diagram of JPEGs prepared with different concentrations of NH_2_‐PEG‐NH_2_ in the water phase and SiO_2_ NPs in the oil phase. If not specified, the oil phase is camellia oil and the oil ratio is 40 vol%. If not specified, the NH_2_‐PEG‐NH_2_ concentration in the water phase is 10 mg mL^−1^ and the SiO_2_ concentration in the oil phase is 20 mg mL^−1^.

In a typical system of JPEGs, the SiO_2_ concentration in the oil phase is 20 mg mL^−1^ and the NH_2_‐PEG‐NH_2_ concentration in the water phase is 10 mg mL^−1^. Since SiO_2_ NPs generally contain hydrophilic ‐OH groups, they tend to stabilize the water/oil interface and reduce the interfacial tension from 22.4 to 18.0 mN m^−1^, as shown by the measurements of dynamic interfacial tensions in **Figure** **2**a. Similarly, NH_2_‐PEG‐NH_2_ could also slightly reduce the interfacial tension. SiO_2_ NPs are negatively charged due to the ionization of ‐O^−^ groups, while NH_2_‐PEG‐NH_2_ molecules are positively charged due to the ionization of NH^4+^ groups. Therefore, each positively‐charged NH_2_‐PEG‐NH_2_ molecule could bind with two negatively‐charged SiO_2_ NPs via electrostatic interaction, forming a cross‐linked network at the water/oil interface. The cross‐linked network of SiO_2_ NPs by NH_2_‐PEG‐NH_2_ is evidenced by the buckling of the cross‐linked interface when the inner fluid is withdrawn, as shown in Figure 2b. JPEGs are different from attractive Pickering emulsion gels (APEGs),^[^ ^6a^ ^]^ in which neighboring NP‐stabilized droplets are bridged by telechelic polymers. No attractive force is observed in the JPEG system when two NP‐stabilized water droplets separate from each other, as shown in Figure 2c. Even when one NP‐stabilized water droplet squeezes another NP‐stabilized water droplet, they are stable against coalescence, compression, and deformation, as shown in Figure 2d. This is because NPs at the water/oil interface are cross‐linked by telechelic polymers and the NP‐stabilized water droplets are very stable, as modeled in the inset of Figure 2d.

In the formation of JPEGs, both SiO_2_ NPs and NH_2_‐PEG‐NH_2_ are essential, as shown in Figure 2e. In the absence of SiO_2_ NPs in the oil phase or NH_2_‐PEG‐NH_2_ in the water phase, no JPEGs could be formed and only water‐in‐oil emulsions are observed, as shown in Figure S1 (Supporting Information). For example, the water‐in‐oil Pickering emulsions prepared with SiO_2_ NPs in the oil phase but no NH_2_‐PEG‐NH_2_ in the water phase are flowable with the viscous modulus G″ larger than the elastic modulus G′, as shown in Figure S2 (Supporting Information). When the molecular weight of NH_2_‐PEG‐NH_2_ decreases from MW≈8000 Da to MW≈1000 Da, JPEGs could still be obtained. Both JPEGs prepared with MW≈1000 Da NH_2_‐PEG‐NH_2_ and MW≈8000 Da NH_2_‐PEG‐NH_2_ are stable after storage at 4 °C for 10 days, as shown in Figure S3 (Supporting Information). JPEGs could be obtained only when the SiO_2_ concentration is ≥15 mg mL^−1^ and the NH_2_‐PEG‐NH_2_ concentration is ≥2 mg mL^−1^.

### 2.3 Characterization of JPEGs

**Figure 3:**
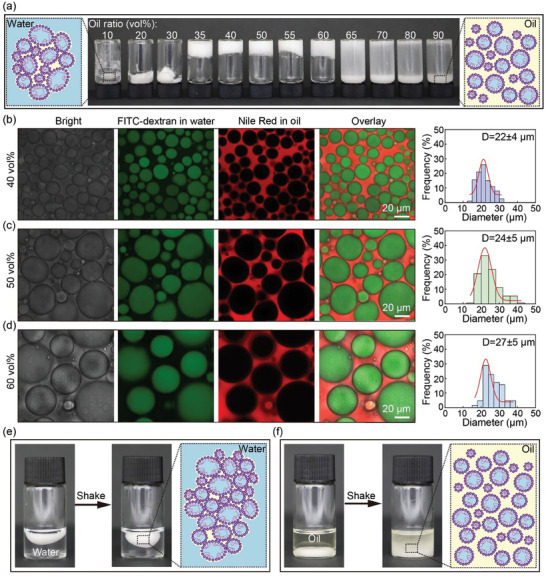
Characterizations of JPEGs prepared with different oil ratios. (a) Photographs of the systems prepared with different oil ratios. JPEGs are formed with oil ratio in the range between 35% and 60 vol%. Fluorescent confocal microscope images and droplet size distributions of JPEGs prepared with (b) 40 vol%, (c) 50 vol%, and (d) 60 vol% oil. (e) JPEGs are stable in water under gentle shaking. (f) JPEGs become unjammed in oil and NP‐stabilized water droplets separate from each other under gentle shaking, as there is no attractive interaction between them.

In addition to the concentrations of SiO_2_ NPs and NH_2_‐PEG‐NH_2_, the oil ratio also plays an important role in the formation of JPEGs, as shown in **Figure** **3**a. Only when the oil ratio is in the range between 35% and 60%, water droplets are fully emulsified and there is no excess oil outside the interstitial volume between NP‐stabilized water droplets, thus leading to stable JPEGs, as shown in Figure S4 (Supporting Information). When the oil ratio is too small, e.g. oil ratio<35%, water droplets cannot be fully emulsified, as shown in Figure S5a (Supporting Information). When the oil ratio is too large, e.g. oil ratio>60%, there is excess oil in the continuous phase and no JPEGs are formed, since NP‐stabilized water droplets are dispersed in the oil phase instead of jammed, as shown in Figure S5b (Supporting Information). JPEGs are different from high internal phase emulsions (HIPEs). The jamming and thus gelling of SiO_2_ NP‐stabilized emulsions in HIPEs typically occurs when the volume ratio of the dispersed phase exceeds 80 vol%. This is because SiO_2_ NP‐stabilized droplets are still susceptible to deformation. However, when SiO_2_ NPs at the water/oil interface are cross‐linked by NH_2_‐PEG‐NH_2_, the cross‐linked NP‐stabilized droplets become hard to deform and the jamming and thus gelling of JPEGs could occur at a low volume ratio of the dispersed phase of 40 vol%.

To observe the microstructure of JPEGs clearly under fluorescent confocal microscope, Nile Red and FITC‐dextran, which serve as fluorescent indicators,^[^ ^7b^ ^]^ are added to the oil and water phases, respectively. The fluorescent confocal microscope images confirm that the JPEG system is consisted of NP‐stabilized water droplets jammed in the oil phase, as water droplets are slightly deformed, and the average size of water droplets increases from 22  to 27 µm, as the oil ratio increases from 40% to 60 vol%, as shown in Figure 3b–d.

The method of JPEGs is versatile and JPEGs can be prepared with different oils, such as camellia oil, olive oil, and safflower oil, as shown in Figure S6 (Supporting Information). In addition, other cationic polymers also support emulsion jamming. JPEGs are successfully prepared with chitosan quaternary ammonium salt, chitosan, and polyethyleneimine (PEI) using the same strategy as JPEGs with NH_2_‐PEG‐NH_2_, as shown in Figure S7 (Supporting Information). Since JPEGs are consisted of jammed NP‐stabilized water droplets in the oil phase, they are stable when floating in water and against gentle shaking, as the JPEG system still keeps as an integrity, as shown in Figure 3e and modeled in the inset. However, JPEGs become unjammed when submerged in oil and NP‐stabilized water droplets separate from each other under gentle shaking, as there are no NH_2_‐PEG‐NH_2_ molecules bridging neighboring NP‐stabilized water droplets and thus there is no attractive interaction between them, as shown in Figure 3f and modeled in the inset.

### 2.4 Viscoelastic Properties of JPEGs

**Figure 4:**
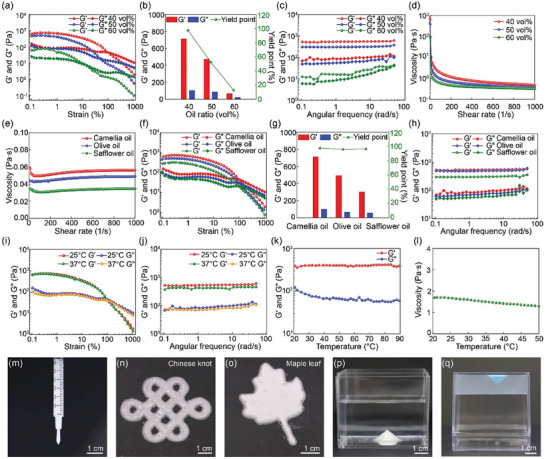
Viscoelastic properties of JPEGs. (a) Strain sweeps of the elastic modulus G′ and viscous modulus G″ of JPEGs prepared with 40%, 50%, and 60 vol% oil, showing characteristic shear‐thinning behaviors. If not specified, the frequency is kept constant at 1 rad/s. (b) Dependance of elastic modulus G′, viscous modulus G″, and yield point on oil ratio. (c) Frequency sweeps of the elastic modulus G′ and viscous modulus G″ of JPEGs prepared with different oil ratios. If not specified, the strain is kept constant at 1%. (d) Apparent viscosities of JPEGs prepared with different oil ratios. If not specified, 40 vol% oil is chosen for subsequent tests. (e) Apparent viscosities of camellia oil, olive oil, and safflower oil. (f) Strain sweeps of the elastic modulus G′ and viscous modulus G″ of JPEGs prepared with different oils, showing characteristic shear‐thinning behaviors. (g) Dependance of elastic modulus G′, viscous modulus G″, and yield point on oil type. (h) Frequency sweeps of the elastic modulus G′ and viscous modulus G″ of JPEGs prepared with different oils. (i) Strain sweeps of the elastic modulus G′ and viscous modulus G″ measured at 25 and 37 °C. (j) Frequency sweeps of the elastic modulus G′ and viscous modulus G″ measured at 25 and 37 °C. (k) Dependance of elastic modulus G′ and viscous modulus G″ on temperature. (l) Dependance of apparent viscosity on temperature. If not specified, experiments are performed at 25 °C. The shear rate is kept constant at 100 1/s. 3D printing of JPEGs (m–o) in air, (p) in water, and (q) in hydrogel.

Viscoelastic properties are important for the applications of JPEGs.^[^ ^10^, ^23^ ^]^ Because NP‐stabilized water droplets are jammed in the system, the elastic modulus G′ is larger than the viscous modulus G″ and JPEGs behave as elastic gels at low shear strain (<10%). However, JPEGs become flowable at high shear strain (>100%), above which NP‐stabilized water droplets could slide along each other, showing characteristic shear‐shinning behaviors, as shown in **Figure** **4**a. As the oil ratio increases, the water droplets become larger and their packing becomes looser, making them more susceptible to flow; therefore, the elastic modulus G′, viscous modulus G″, and yield point decrease, as shown in Figure 4b. The frequency sweeps are measured in the linear viscoelastic region^[^ ^24^ ^]^ and the results suggest that the elastic modulus and viscous modulus show little dependence on the frequency and time, as shown in Figure 4c and Figure S8 (Supporting Information), respectively. The apparent viscosities of JPEGs prepared with different oil ratios decrease as the oil ratio increases, as shown in Figure 4d.

The viscoelastic properties of JPEGs prepared with different oils are also investigated. The apparent viscosities of camellia oil (0.055 Pa·s), olive oil (0.048 Pa·s), and safflower oil (0.034 Pa·s) gradually decrease, as shown in Figure 4e. As the oil viscosity decreases, the elastic modulus G′ and viscous modulus G″ decrease while the yield point barely changes, since the oil ratio is kept constant at 40 vol%, as shown in Figure 4f,g. Similarly, the elastic modulus and viscous modulus of JPEGs prepared with different oils show little dependance on the frequency, as shown in Figure 4h. In addition, stain sweeps, frequency sweeps, and time sweeps of the elastic modulus G′ and viscous modulus G″ show little difference when measured at 25 and 37 °C, as shown in Figure 4i,j and Figure S9 (Supporting Information), respectively. Overall, as the temperature increases, the elastic modulus G′ barely changes, while the viscous modulus G″ and viscosity slightly decrease, as shown in Figure 4k,l and Figure S10 (Supporting Information).

The shear‐thinning viscoelastic property and excellent stability make JPEGs an ideal ink for 3D printing. JPEGs could flow like a liquid in the channel when infused under external pressure and maintain their shape when squeezed out of the nozzle, as shown in Figure 4m. Combined with the dedicated control of 3D printing, JPEGs could be printed into different patterns and shapes, as shown in Figure 4n,o. In addition, because of the characteristics of water‐in‐oil emulsions, JPEGs could also be printed in water and hydrogel, as shown in Figure 4p,q, respectively.

### 2.5 Stability and Release of JPEGs

**Figure 5:**
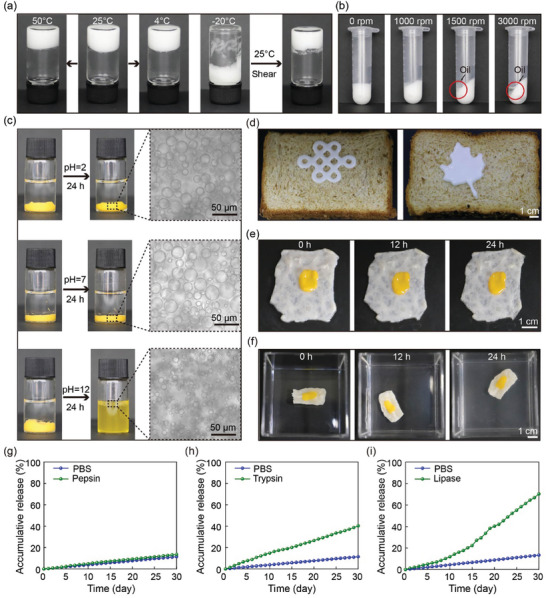
Stability and release of JPEGs. (a) Stability of JPEGs at different temperatures. JPEGs are destroyed at −20 °C due to ice crystallization and could be reconstructed at 25 °C by shearing. (b) Stability of JPEGs against different centrifugation forces. The gel structure is stable against centrifugation force up to 3000 rpm, though some oil is squeezed out at 1500 rpm. (c) Stability of JPEGs in water at different pHs, e.g. pH 2, 7, and 12. The yellow color is attributed to FITC‐dextran loaded in JPEGs. (d) Pastes of JPEGs printed on bread. (e) JPEGs can well adhere to fresh intestinal tissues. (f) Stability of JPEGs adhered to fresh intestinal tissues in water. (g) Release of JPEGs in PBS and pepsin (simulated gastric liquid) at pH 1.5 and T = 37 °C. (h) Release of JPEGs in PBS and trypsin (simulated intestinal liquid) at pH 6.8 and T 37 °C. (i) Release of JPEGs in PBS and lipase (simulated intestinal liquid) at pH 7.4 and T = 37 °C. Data represent means ± standard error (*n* = 3).

JPEGs are stable over a wide temperature range from 4 to 50 °C, as shown in **Figure** **5**a. No observable changes are observed in JPEGs after 15 days at 4 °C (Figure S11, Supporting Information) or after 5 months at 25 °C (Figure S12, Supporting Information), suggesting a good storage stability. In addition, the droplet sizes of JPEGs stored at 4 and 25 °C remains unchanged after 15 days, as measured and shown in Figures S13 and S14 (Supporting Information), respectively. JPEGs are destroyed at −20 °C, as the oil layer between neighboring water droplets could be very thin and the volume of water droplets increases when they crystallize into ice, thus destroying the structure of JPEGs. JPEGs could be reformed at room temperature simply by vortexing. JPEGs are also stable against centrifugation up to 3000 rpm, even though a small amount of oil is squeezed out by compressing water droplets above 1500 rpm, as shown in Figure 5b.

When JPEGs stick at the bottom and are submerged in water at pH between 2 and 7, no leakage of FITC‐dextran from JPEGs is observed, suggesting that JPEGs are stable at low pH, as shown in Figure 5c. However, at high pH, e.g. pH 12, JPEGs are gradually destroyed, releasing FITC‐dextran to the water phase. This is because in the presence of abundance OH^−^ groups at high pH, NH_2_‐PEG‐NH_2_ could not be ionized to form positively‐charged NH_4_^+^ groups and the electrostatic interactions between NH_2_‐PEG‐NH_2_ molecules and SiO_2_ NPs are suppressed, thus destabilizing the JPEG system.

Emulsion gels are widely used in daily life. The JPEG system provides a versatile way to prepare oil‐in‐water emulsion gels, which are hard to be achieved by conventional methods. Since JPEGs are consisted of biocompatible materials and possess viscoelastic properties for easy processing, JPEGs could be used in food and developed into bread pastes, which could reduce the amount of oil used and be printed into various delicate patterns, as shown in Figure 5d.

JPEGs are also excellent candidates of injectable and adhesive delivery systems. Hydrophilic and hydrophobic drugs could be loaded in the water and oil phases, respectively. For example, FITC‐dextran and Nile Red are used model hydrophilic and hydrophobic drugs and loaded in the water and oil phases, respectively. Drug‐loaded JPEGs show good adhesion to fresh wet intestinal mucosa, which may be attributed to the good deformability and good contact of JPEGs with wet tissues as JPEGs are smeared onto the wet tissues, as shown in Figure 5e, and good stability in water, as shown in Figure 5f. To test the release of model drugs from JPEGs, the accumulative release of FITC‐dextran from JPEGs is determined from its absorption peak at 495 nm as measured by UV–vis adsorption. In simulated gastric liquids, e.g. pepsin, the model drug is barely released from JPEGs and there is no difference between pepsin and PBS, as shown in Figure 5g. In contrast, the model drug is continuously released from JPEGs in simulated intestinal liquids, e.g. trypsin and lipase, which can gradually decompose the continuous oil phase, thus releasing the encapsulated cargo in a sustained manner, as shown in Figures 5h,I. As the continuous oil phase is gradually hydrolyzed by trypsin and lipase, JPEGs gradually disintegrate, as shown in Figure S15 (Supporting Information). These results suggest that JPEGs are well suited for controlled drug delivery applications in tissue engineering.

The designed JPEG system has many advantages, including i) The strategy to prepare JPEGs is versatile and applicable to different oil phases, different cationic polymers, and a wide range of oil ratios. ii) The JPEG system is biocompatible and could be applied in various areas. iii) The JPEG system possesses interesting viscoelastic properties and its shear‐thinning behavior makes it easy for processing. iv) JPEGs are stable over a wide temperature range and against centrifugation. v) Hydrophilic and hydrophobic drugs could simultaneously be loaded in the JPEG system and released in a sustained manner by external stimuli. vi) JPEGs could be developed into drug delivery systems with good adhesion to wet tissues, especially good for tissue engineering.

## 3 Conclusion

In this study, a new strategy is developed to prepare emulsion gels by jamming interface‐crosslinked NP‐stabilized water droplets in the continuous oil phase. In the JPEG system, water droplets are stabilized by negatively‐charged SiO_2_ NPs, which are cross‐linked by positively‐charged NH_2_‐PEG‐NH_2_ molecules, making water droplets hard to deform and thus forming jammed emulsion gels. The strategy to prepare JPEGs is applicable to diverse oil phases and a wide range of oil ratios. The designed JPEGs possess many advantages, including good biocompatibility for widespread applications, shear‐thinning rheological property for easy processing, good stability over a wide temperature range and against centrifugation, good adhesion to wet tissues for tissue engineering, and well‐controlled sustained release under intestinal conditions. The strategy to achieve emulsion gels is different from previous methods and provides a simple and effective platform for the design of new materials, which will definitely inspire more innovations.

## Conflict of Interest

The authors declare no conflict of interest.

## Supporting information



Supporting Information

## Data Availability

The data that support the findings of this study are available in the supplementary material of this article.
