# Supplementary material for: Jammed Pickering Emulsion Gels
Source: Adv Sci (Weinh). 2024 Nov 14;11(48):2409678. doi: 10.1002/advs.202409678 (PMC11672259; doi:10.1002/advs.202409678)
Supplement: Supplementary file 1 — Supporting Information [file ADVS-11-2409678-s001.docx]

Supporting Information

Jammed Pickering Emulsion Gels

Jia Zhang, Yuan Zheng, Baoling Guo, Dongpeng Sun, Yao Xiao, Ze Yang, Rongrong Liu, Jingyi Chen, Baiheng Wu, Peng Zhao, Jian Ruan, David A Weitz and Dong Chen^*^

**Materials and Methods**

**Materials**

Polyoxyethylene bis(amine) (NH_2_-PEG-NH_2_, MW~1000 Da and MW~8000 Da), FITC-dextran (MW~10000 Da), pepsin (>3000 U mg^-1^), trypsin and lipase (20000 U g^-1^) are purchased from Macklin. White carbon black (Hydrophobic SiO_2_) is purchased from Shandong Yousuo Chemical Technology Corporation. Camellia oil is purchased from Guangdong Youfeng Oil Tea Technology Corporation. Olive oil is purchased from Anhui Chugu Food Corporation. Safflower oil is purchased from Goodbe Work Shop. Nile Red and chitosan quaternary ammonium salt (Degree of substitution~90%) are purchased from Shanghai Yuanye Bio-Technology Corporation. Chitosan (low molecular weight) is purchased from Sigma-Aldrich. Poly(ethylene imine) (PEI, MW~600 Da) is purchased from Aladdin. Deionized (DI) water is used throughout the study.

**Methoads**

**Preparation of** **jammed Pickering emulsion gels (JPEGs)**

To prepare JPEGs, hydrophobic SiO_2_ nanoparticles (NPs) are dispersed in the oil phase, and polyoxyethylene bis(amine) (NH_2_-PEG-NH_2_) is dissolved in the water phase. When the two phases are emulsified by votexing, SiO_2_ NPs serve as colloidal surfactants and stabilize water droplets, forming Pickering emulsions. Meanwhile, each positively-charged NH_2_-PEG-NH_2_ in the water phase binds with two negatively-charged SiO_2_ NPs at the interface, forming a cross-linked network and making water droplets hard to deform. During the emulsification process, water droplets are sheared into smaller droplets and the interstitial volume between water droplets becomes smaller, eventually forming JPEGs after a couple of minutes in stationary. The oil phase could be camellia oil, olive oil or safflower oil. The SiO_2_ concentration is ≥15 mg mL^-1^ and the NH_2_-PEG-NH_2_ concentration is ≥2 mg mL^-1^. The oil volume ratio could be between 35% and 60%.

**Measurement of interfacial tensions**

The interfacial tensions of water in oil are measured by pendent drop experiments at room temperature (Drop-Meter A-200, Ningbo NB Scientific Instruments Corporation). The interactions between two NP-stabilized water droplets in the camellia oil phase are explored by a tensiometer (DCAT 25, DataPhysics Instruments). The water phase contains 10 mg mL^-1^ NH_2_-PEG-NH_2_ and the camellia oil phase contains 20 mg mL^-1^ SiO_2_ NPs. One water droplet is placed at the bottom. The other water droplet is suspended on the top. The bottom droplet gradually approaches the top droplet and squeezes it at a speed of 0.01 mm s^-1^. The force-displacement curves are recorded during the process.

**Fluorescent confocal microscope images of JPEGs**

To determine the emulsion type of JPEGs, the oil phase is dyed with Nile Red and the water phase is dyed with FITC-dextran. The JPEGs are then imaged using a fluorescent confocal microscope (TCS SP5 Ⅱ, Leica). FITC-dextran is excited by an Argon laser with an excitation wavelength of 488 nm and Nile Red is excited by a HeNe laser with an excitation wavelength of 543 nm. The green fluorescence emitted by FITC-dextran is detected by a channel collecting 500-520 nm signals and the red fluorescence emitted by Nile Red is detected by a separate channel collecting 650-800 nm signals.

**Rheological measurements of JPEGs**

The viscoelastic properties of JPEGs are measured by a rotating rheometer (MARS 60, HAAKE) equipped with two parallel flat plates (Diameter=20 mm). The gap between the two plates is fixed at 1 mm. The JPEGs used for rheological measurements are prepared using 10 mg mL^-1^ NH_2_-PEG-NH_2_ in water and 20 mg mL^-1^ SiO_2_ NPs in the camellia oil phase. The oil volume ratio is 40%, 50%, and 60%. A solvent-trapping device is placed above the plates to avoid the evaporation of JPEGs.

Strain sweeps of the elastic modulus G′ and viscous modulus G″ of JPEGs are measured from 0.1 to 1000% at a constant frequency of 1 rad/s at 25 and 37 °C. Frequency sweeps of the elastic modulus G′ and viscous modulus G″ of JPEGs are measured from 0.1 to 50 rad/s at a constant strain of 1% at 25 and 37 °C. Temperature sweeps of the elastic modulus G′ and viscous modulus G″ are measured from 20 to 90 ℃ at a constant frequency of 1 rad/s and a constant strain of 1%. Time sweeps are measured at a constant frequency of 1 rad/s and a constant strain of 1% at 25 °C. The viscosity of JPEGs is measured from 0.1 to 1000 1/s at 25 and 37°C. The dependence of viscosity on temperature is measured from 20 to 50°C at a constant shear rate of 100 1/s.

**Stability tests of JPEGs**

Prepared JPEGs are placed at different temperatures, e.g. -20, 4, and 50 ℃, for 12 hours to test their thermal stability. To test their stability against centrifugation, JPEGs are centrifuged at different rotational speeds, e.g. 1000, 1500, and 3000 rpm, for 5 minutes. The stability of JPEGs is also tested at different pHs, e.g. pH=2, pH=7, and pH=12. The FITC-dextran released from JPEGs is quantitatively determined by UV-Vis measurements.

**3D printing of JPEGs**

Since JPEGs possess the typical shearing-thinning viscoelastic property, JPEGs could be used as an ink for 3D printing, which could be extruded out of the nozzle and maintain its shape after leaving the nozzle. 2D patterns and 3D structures are first designed and decoded into continuous printing paths by a gcode. Then, 2D patterns and 3D structures of JPEGs are directly printed in air and water.

**Enzyme degradation of JPEGs**

Fresh intestinal tissues bought from market are washed with PBS before use. To test the adhesion, JPEGs are placed on the wet intestinal mucosa surface.

The stability of JPEGs in pepsin (10 g L^-1^) at pH=1.5, in trypsin (10 g L^-1^) at pH=6.8 and in lipase (5 g L^-1^) at pH=7.4 is tested as follows. 0.5 mL JPEG is added into 1 mL enzyme solution. To test the release profile, FITC-dextran is used as the model hydrophilic active and loaded in JPEGs. The concentration of released FITC-dextran in the enzyme solution is determined by measuring its absorption peak at 495 nm using a UV-Vis spectrometer (UV-1800, Shimadzu). For each day, 1 mL enzyme solution is removed for measurement and replaced with 1 mL fresh enzyme solution. The release kinetics are monitored constitutively over time.

**Table S1.** Acronyms

| Full Name | Acronym |
| --- | --- |
| Jammed Pickering Emulsion Gel | JPEG |
| Nanoparticle | NP |
| Silica | SiO_2_ |
| Polyoxyethylene bis(amine) | NH_2_-PEG-NH_2_ |
| Attractive Pickering Emulsion Gel | APEG |
| High Internal Phase Emulsions | HIPEs |
| Poly(ethylene imine) | PEI |

**Table S2.** Compositions of JPEGs for different experiments

| Oil Type | SiO_2_ in Oil (mg mL^-1^) | NH_2_-PEG-NH_2_ in Water (mg mL^-1^) | Oil Ratio (vol%) | Experiment |
| --- | --- | --- | --- | --- |
| Camellia oil | 0 to 30 | 0 to 10 | 40 | Phase diagram |
| Camellia oil | 20 | 10 | 40 | Fluorescent confocal microscope image;  Rheological test |
| Olive oil |  |  | 50 |  |
| Safflower oil |  |  | 60 |  |
| Camellia oil | 20 | 10 | 40 | Interfacial tension;  3D printing;  Stability test;  Release measurement |


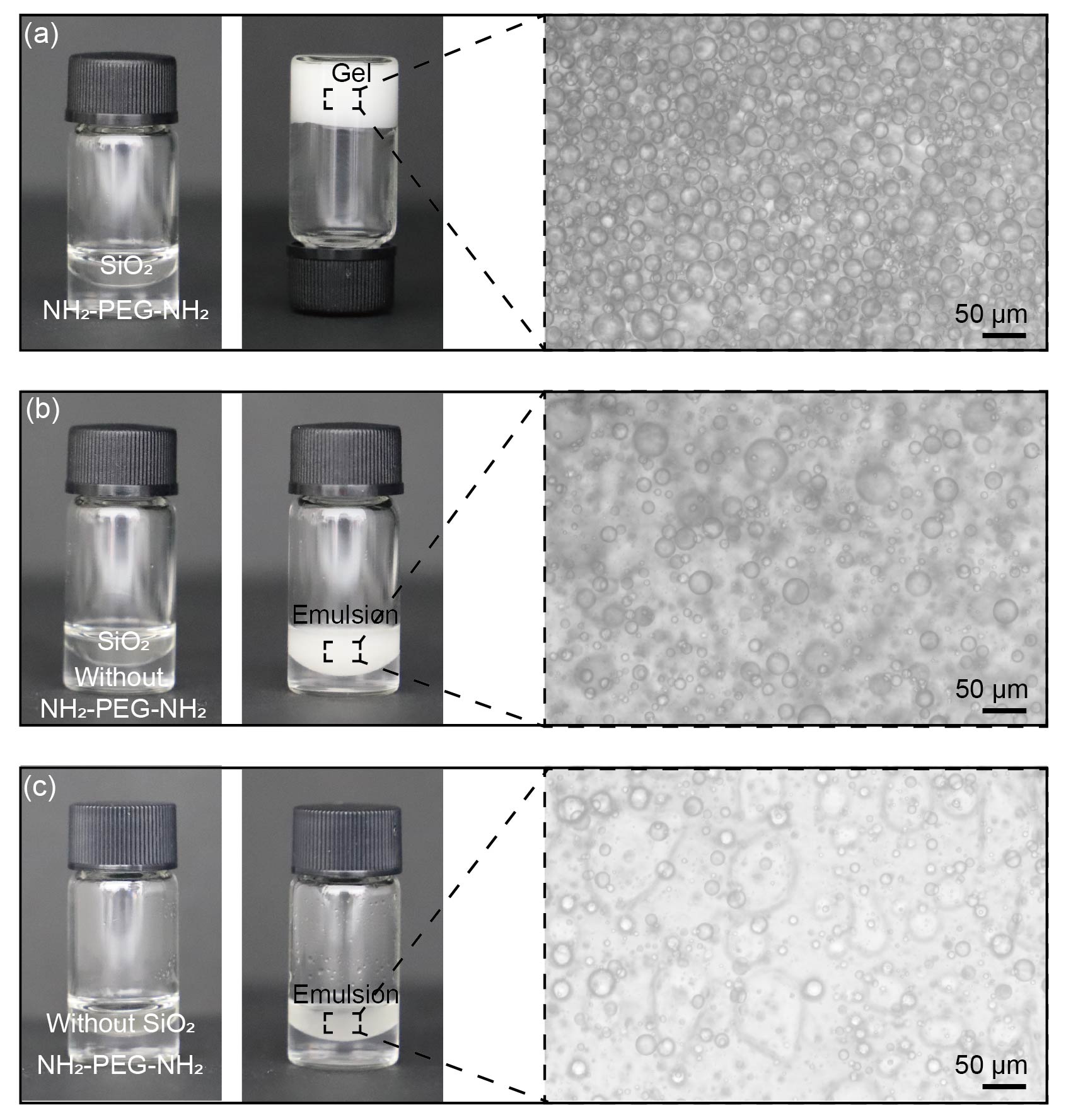


**Figure S1.** Preparation of jammed Pickering emulsion gels (JPEGs). (a) JPEGs are obtained only in the system with hydrophobic SiO_2_ NPs in the oil phase and NH_2_-PEG-NH_2_ in the water phase. (b) Only water-in-oil Pickering emulsions are observed in the system with hydrophobic SiO_2_ NPs in the oil phase but no NH_2_-PEG-NH_2_ in the water phase. (c) Only a few water-in-oil emulsions are observed in the system with NH_2_-PEG-NH_2_ in the water phase but no hydrophobic SiO_2_ NPs in the oil phase. The NH_2_-PEG-NH_2_ concentration in the water phase is 10 mg mL^-1^ and the SiO_2_ concentration in the oil phase is 20 mg mL^-1^. If not specified, the oil volume ratio is 40 vol%. If not specified, camellia oil is used as the oil phase.


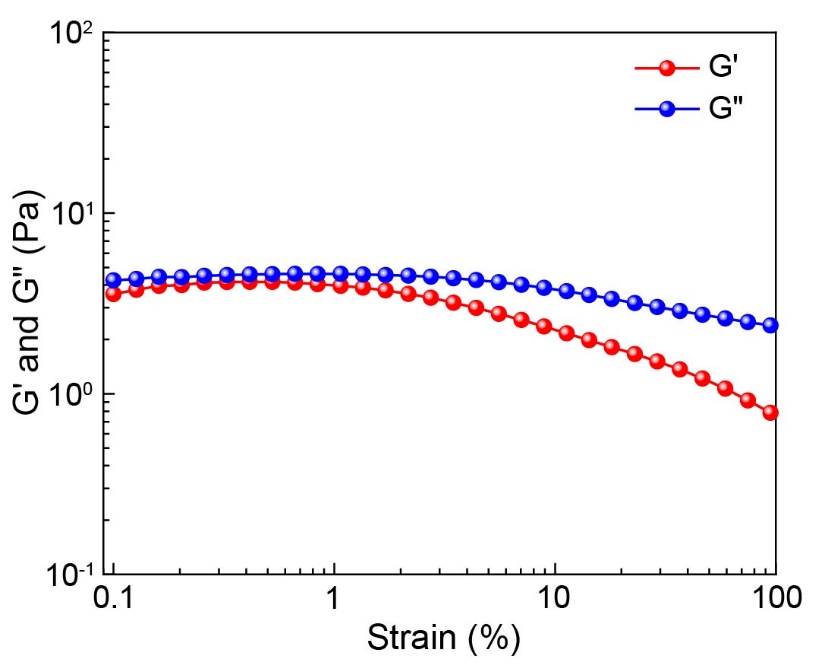


**Figure S2.** Strain sweeps of the elastic modulus G′ and viscous modulus G″ of water-in-oil Pickering emulsions stabilized with hydrophobic SiO_2_ NPs in the oil phase but no NH_2_-PEG-NH_2_ in the water phase.


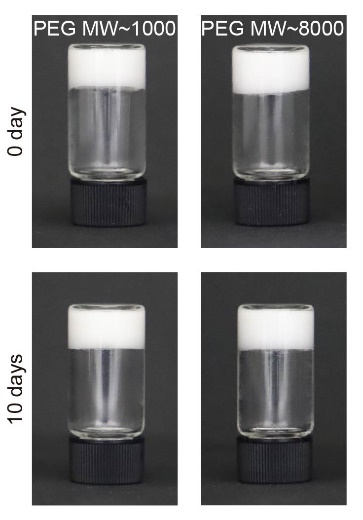


**Figure S3.** JPEGs prepared with MW~1000 Da NH_2_-PEG-NH_2_ or MW~8000 Da NH_2_-PEG-NH_2_. Both JPEGs are stable after storage at 4 ℃ for 10 days. The oil phase is camellia oil and the oil ratio is 40 vol%. The NH_2_-PEG-NH_2_ in the water phase is 10 mg mL^-1^ and the SiO_2_ concentration in the oil phase is 20 mg mL^-1^.


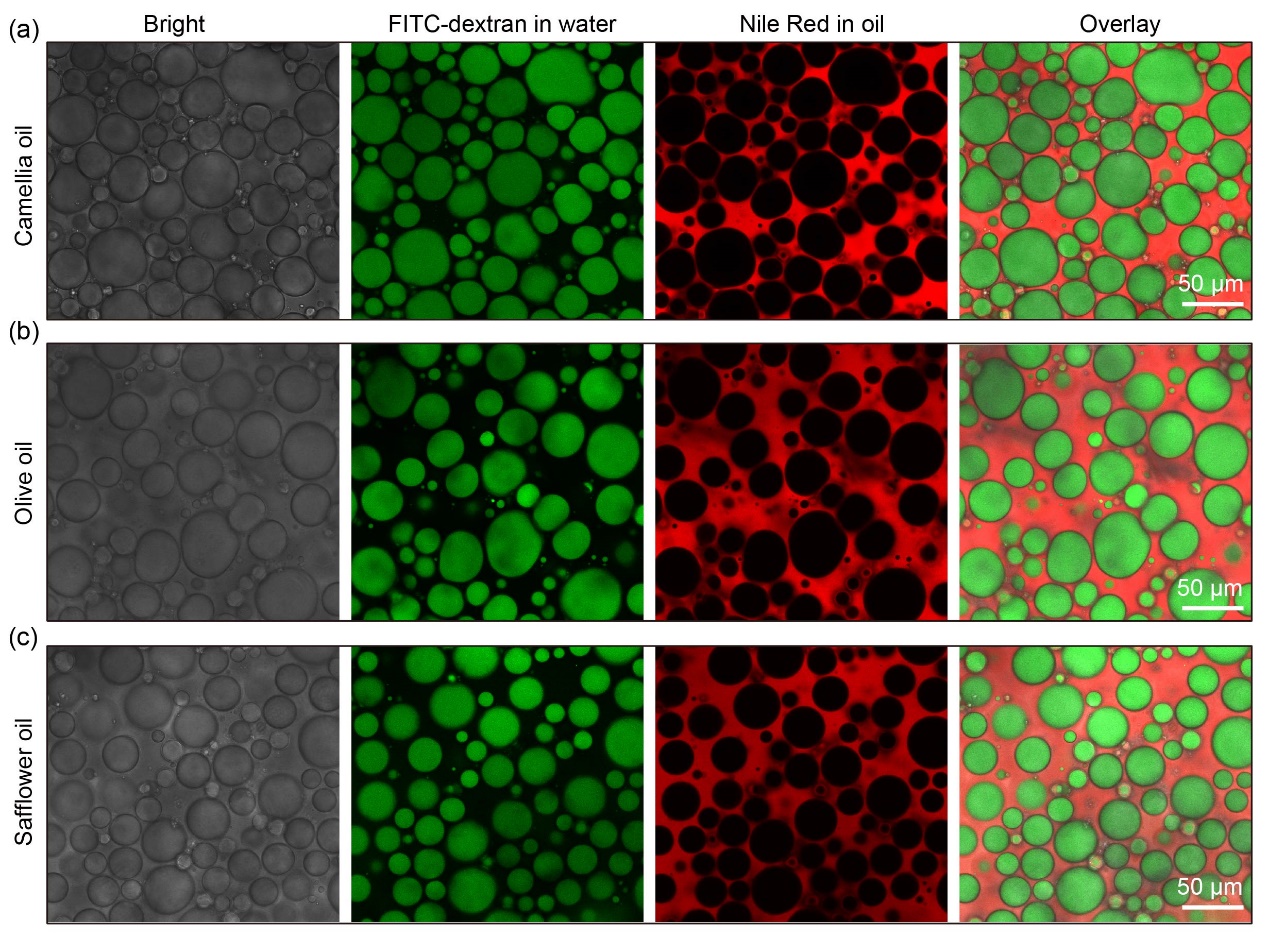


**Figure S4.** Optical and fluorescent confocal microscope images of JPEGs prepared using (a) camellia oil, (b) olive oil, and (c) safflower oil. If not specified, JPEGs are loaded with FITC-dextran in the water phase and Nile Red in the oil phase for fluorescent imaging.


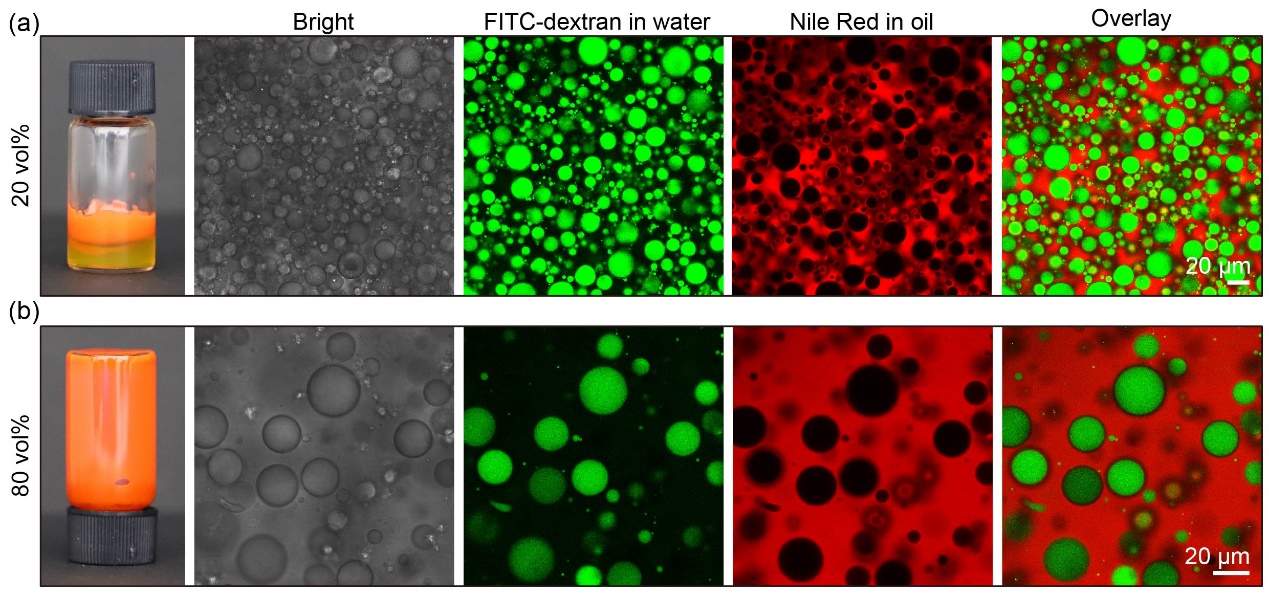


**Figure S5.** Optical and fluorescent confocal microscope images of water-in-oil emulsions prepared with (a) 20 vol% oil and (b) 80 vol% oil.


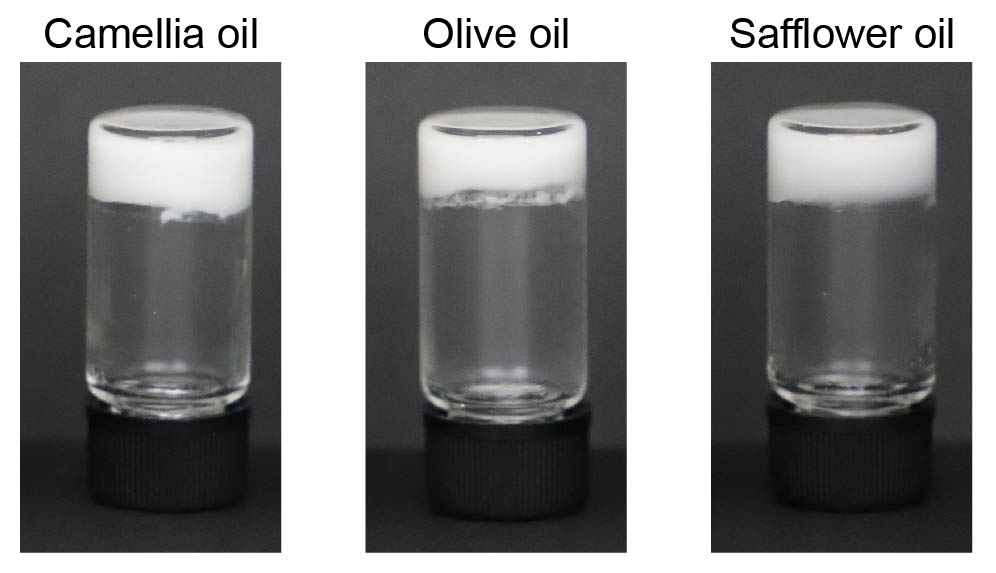


**Figure S6.** JPEGs prepared using camellia oil, olive oil, and safflower oil.


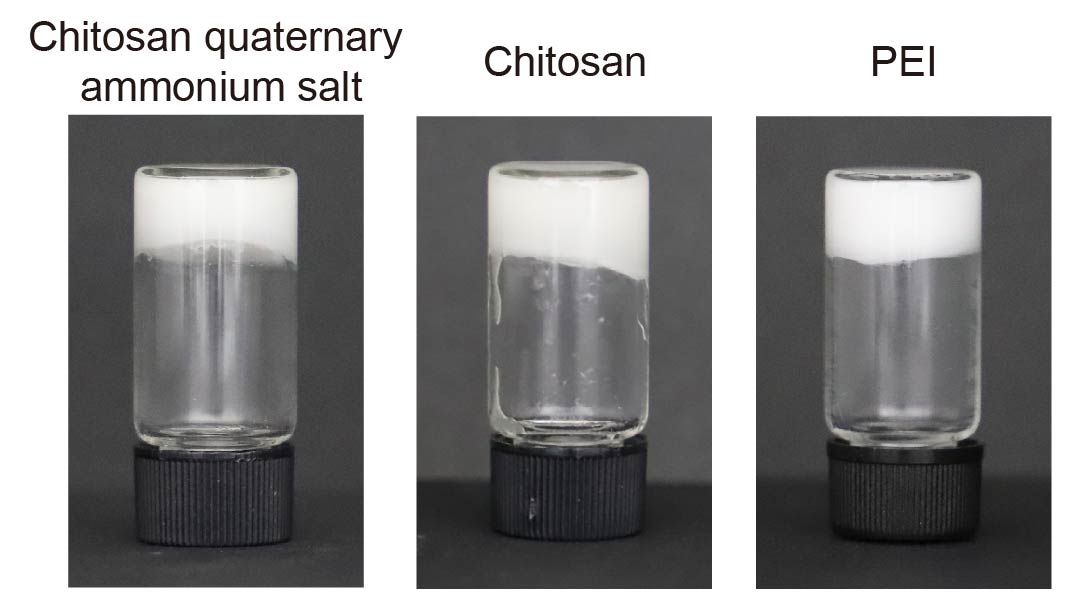


**Figure S7.** JPEGs prepared with cationic polymers, such as chitosan quaternary ammonium salt, chitosan, and PEI, in the water phase. The oil phase is camellia oil and the oil ratio is 40 vol%. The concentrations of chitosan quaternary ammonium salt and PEI in the water phase are 10 mg mL^-1^. The concentration of chitosan dissolved by 2 wt% citric acid in the water phase is 20 mg mL^-1^. The SiO_2_ concentration in the oil phase is 20 mg mL^-1^.


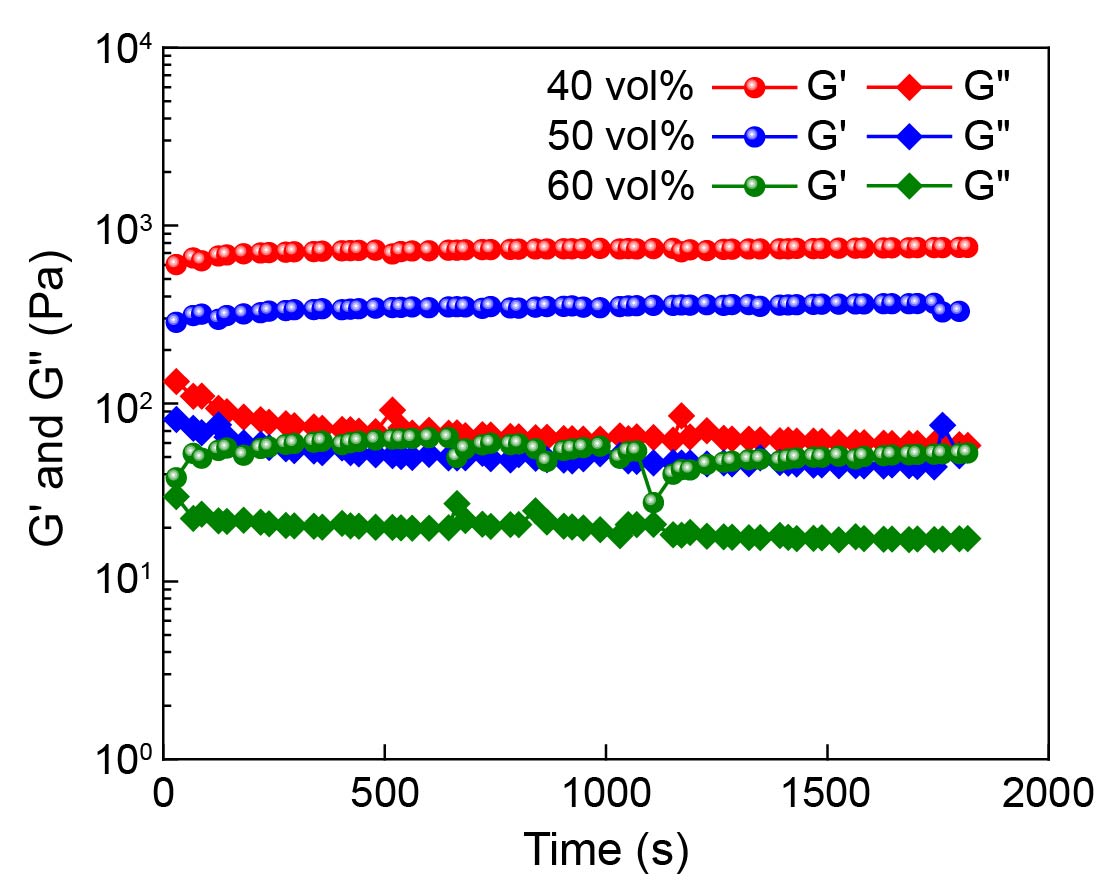


**Figure S8.** Time sweeps of the elastic modulus G′ and viscous modulus G″ of JPEGs prepared with 40 vol%, 50 vol%, and 60 vol% oil. If not specified, the strain is kept constant at 1% and the frequency is kept constant at 1 rad/s. If not specified, the temperature is 25 ℃.


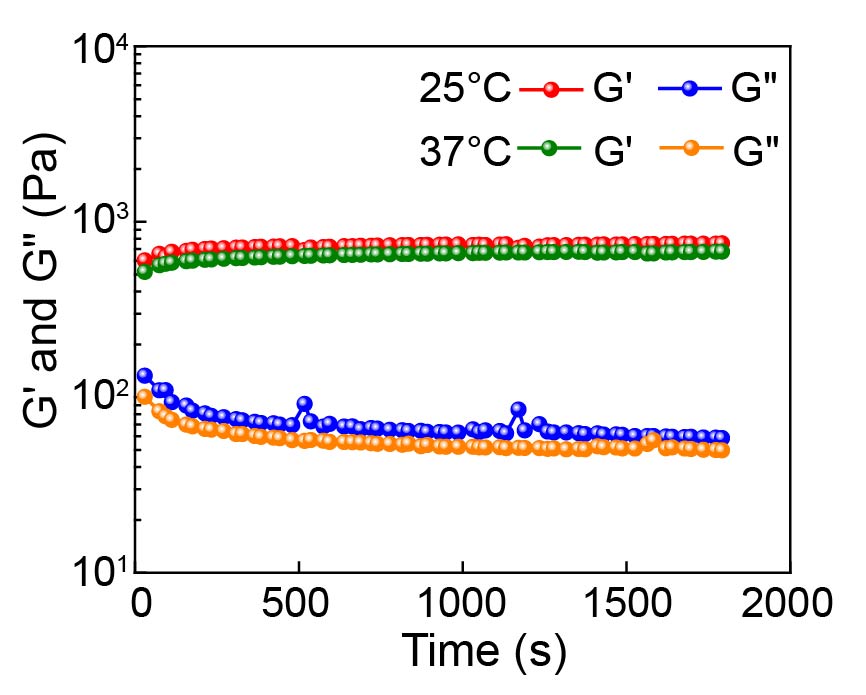


**Figure S9.** Time sweeps of the elastic modulus G′ and viscous modulus G″ of JPEGs measured at 25 and 37 ℃.


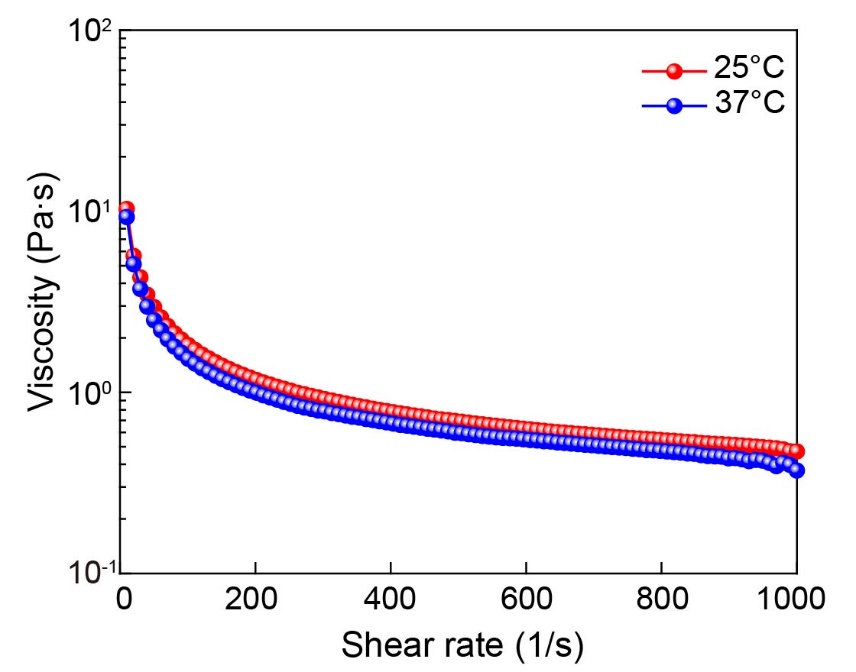


**Figure S10.** Apparent viscosity of JPEGs measured at 25 and 37 ℃.


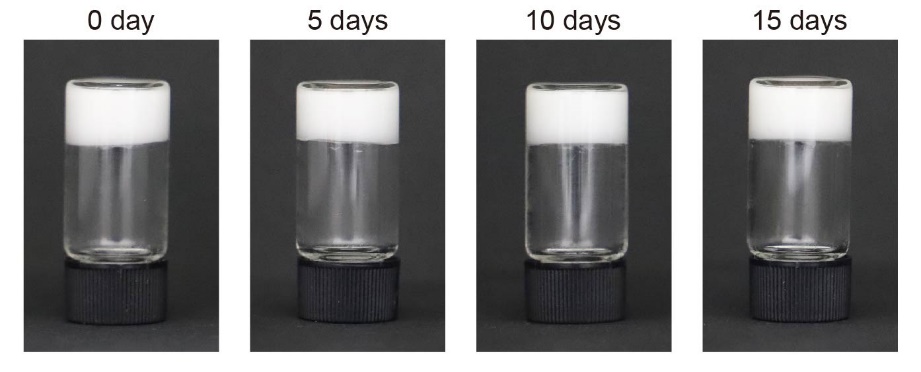


**Figure S11.** Stability of JPEGs at 4 ℃ after 15 days.


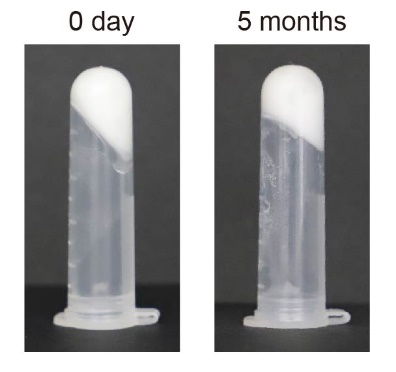


**Figure S12.** Stability of JPEGs at 25 ℃ after 5 months.

**
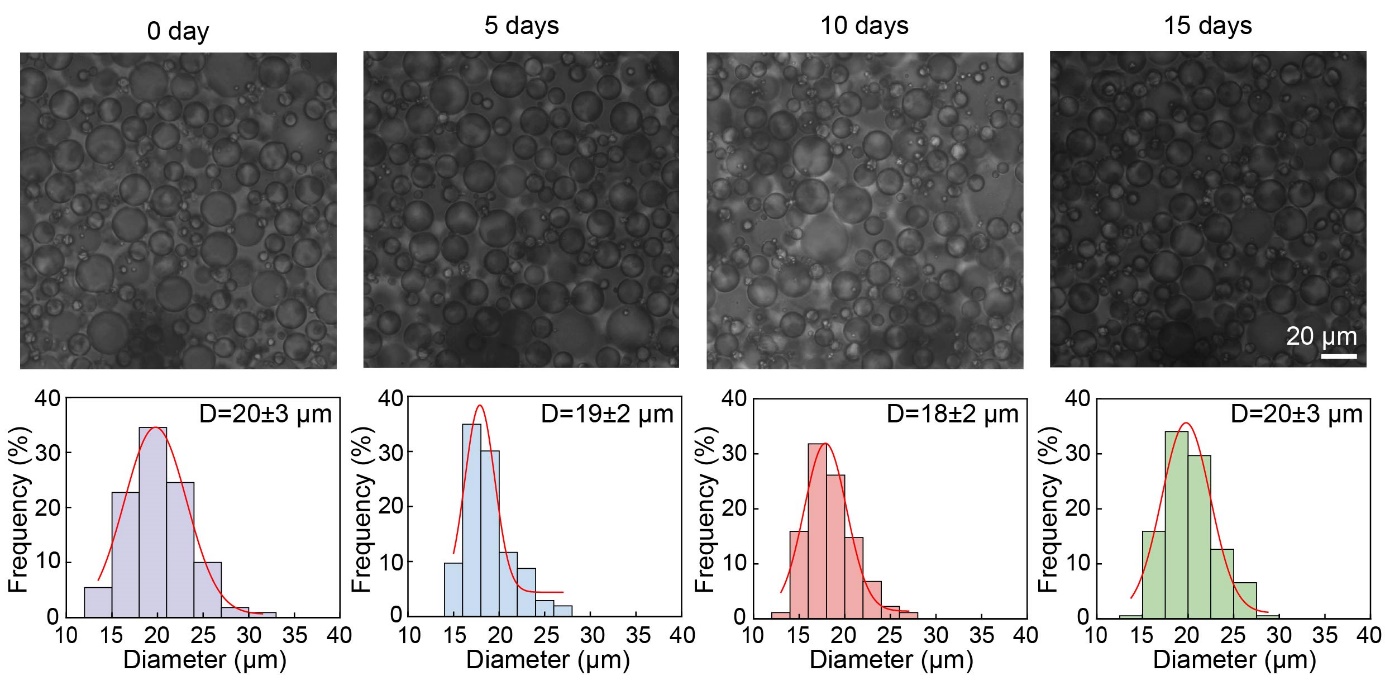
**

**Figure S13.** Optical images and droplet size distributions of JPEGs at 4 ℃ after 15 days.


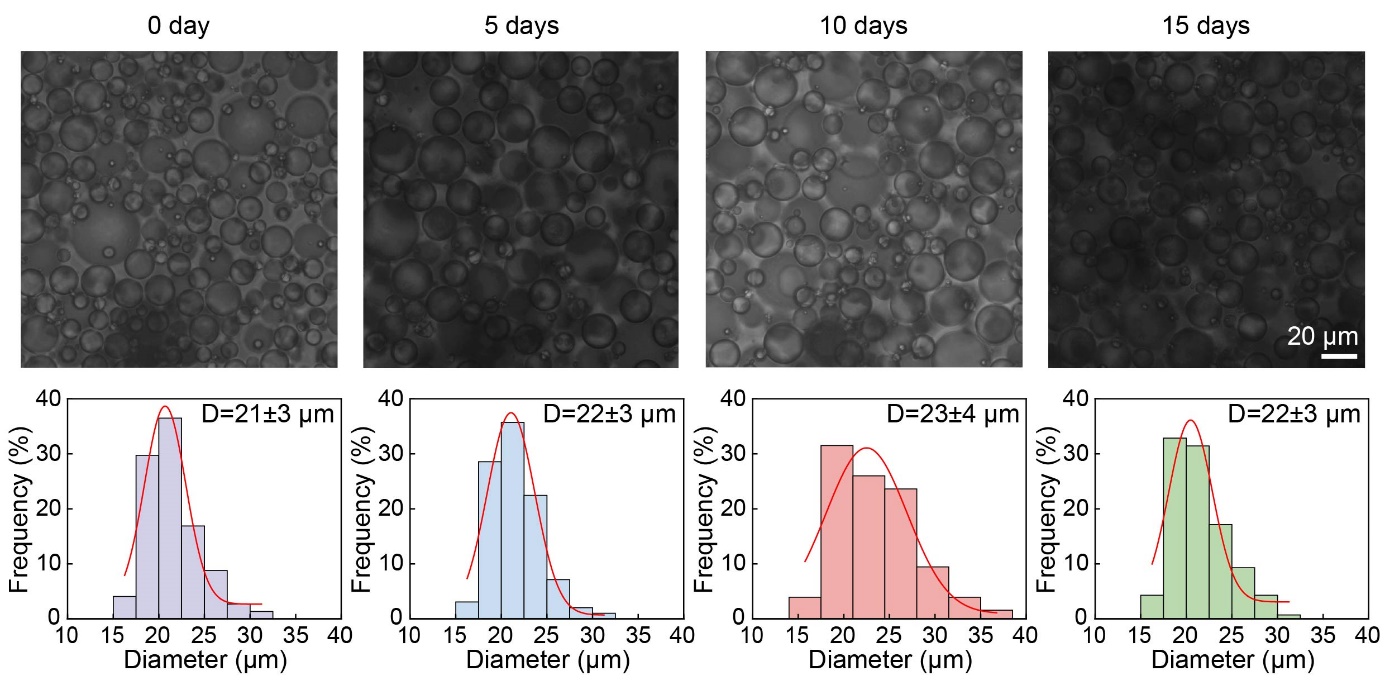


**Figure S14.** Optical images and droplet size distributions of JPEGs at 25 ℃ after 15 days.


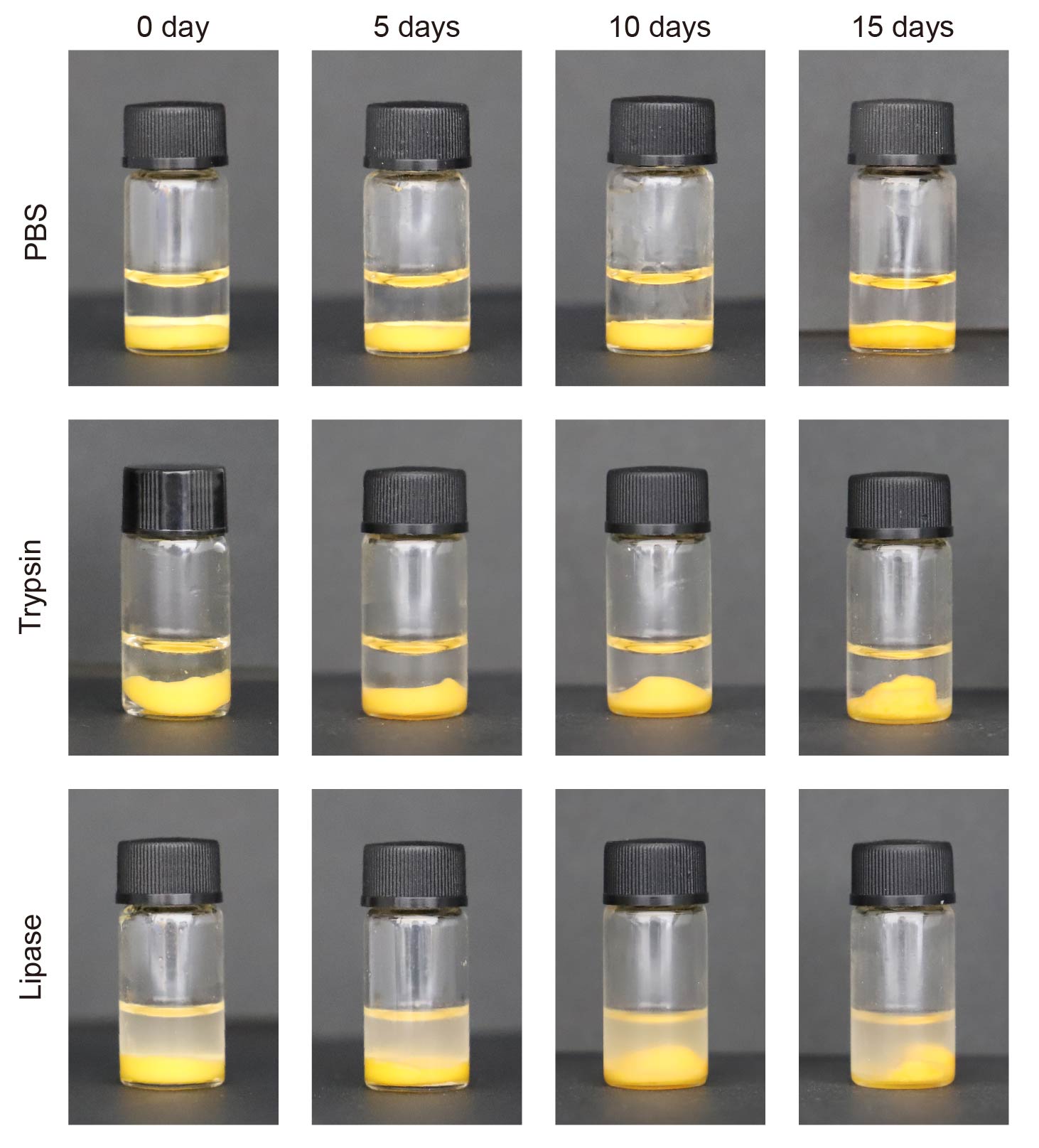


**Figure S15.** Snapshots of JPEGs in PBS, trypsin and lipase over time.
